# Antifungal effectiveness of various intracanal medicaments against *Candida albicans*: an ex-vivo study

**DOI:** 10.1186/1472-6831-14-53

**Published:** 2014-05-13

**Authors:** Eu Gene Chua, Abhishek Parolia, Priya Ahlawat, Allan Pau, Fabian Davamani Amalraj

**Affiliations:** 1 School of Dentistry, International Medical University, No.126, Jalan Jalil Perkasa 19, Bukit Jalil 57000 Kuala Lumpur, Malaysia; 2 School of Human Biology, International Medical University, Kuala Lumpur, Malaysia

**Keywords:** Antifungal, *Candida albicans*, Endodontics, Ex-vivo, Medicaments

## Abstract

**Background:**

To investigate the antifungal activity of propolis, triple antibiotic paste (TAP), 2% chlorhexidine gel and calcium hydroxide with propylene glycol on *Candida albicans*-infected root canal dentinal tubules at two different depths (200 μm and 400 μm) and two time intervals (day 1 and 7).

**Methods:**

A total of 90 extracted human teeth were sectioned below the cementoenamel junction and the apical part of the root to obtain 6 mm of the middle third of the root. The root canal was enlarged to an internal diameter of 0.9 mm using Pesso Reamer size no. 2 (Mani®, UT, Japan), followed by canal irrigation and autoclaved. The specimens were infected for 21 days with *C. albicans*. Then, the specimens were divided into five groups prior to placement of intracanal medicaments. Group 1 (propolis), Group 2 (triple antibiotic paste), Group 3 (2% chlorhexidine Gel), Group 4 (calcium hydroxide with propylene glycol), and Group 5 (sterile saline as negative control). At the end of 1 and 7 days, dentine shavings were collected at two depths into the dentinal tubules (200 μm and 400 μm), and the total numbers of colony forming units were calculated for assessing the remaining vital viable fungal population. The values were analysed statistically using non-paramatric Kruskal-Wallis and Mann–Whitney-U tests to compare the median reduction of *Candida albicans* between all intracanal medicaments. Probability values of P < 0.05 were set as the reference for statistically significant results.

**Results:**

The reduction in number of colony forming units was statistically significant in all groups compared to the control group (sterile saline), except propolis at day 1 (400 μm depth).

**Conclusion:**

Propolis was less effective than triple antibiotic paste, 2% chlorhexidine gel and calcium hydroxide with propylene glycol against *C. albicans* on day 1 at 400 μm deep inside the dentinal tubules, but equally effective after 7 days at both depths.

## Background

Microorganisms are the major causative agents in the development of pulpal and periapical inflammation. It has been shown that periapical lesions heals at a higher rate in teeth in the absence of microbial contamination/infection in the root canal [1]. The primary objective of endodontic treatment is to eliminate microorganisms from the infected root canal system. Chemomechanical instrumentation removes the majority of the microorganisms; however, it is difficult to completely eliminate the microorganisms because of anatomical complexity and the limitation in accessing the canal system by instruments and irrigants [2].

There has been a growing concern about persistent apical periodontitis, in which microorganisms are resistant to routine therapy and the infection may persist despite treatment [3]. Therefore, the need for intracanal medication increases especially in those cases where infection is resistant to the regular treatment and the outcome of endodontic therapy is compromised. Years of microbiological studies on persistent apical periodontitis have shown that *Candida albicans* are the most commonly found fungi, ranging from 7-18% of the infections [4]. *C. albicans* are also encountered as a normal oral flora, hence the presence of *C. albicans* in an infected root canal is highly favorable [5-7].

*C. albicans* retains several virulence factors which can contribute to persistent apical periodontitis. The hyphal formation and thigmotropism property allow *C. albicans* to penetrate deep into the dentinal tubules, and also phenotypic alteration of *C. albicans* helps to adapt in ecologically harsh conditions, as in high alkaline environment [8]. These virulence factors render *C. albicans* to be resistant to calcium hydroxide, which is the most commonly used intracanal medicament [9]. Additionally in the dentinal tubules the buffer substances can prevent calcium hydroxide activity by causing the pH to be lowered drop [10,11].

Calcium hydroxide has limitation, the base water is acidic because it is self-regulating and does not raise the pH, and a variety of agents have been introduced as inter-appointment medicaments. These intracanal medicaments must be able to penetrate deep into the dentinal tubules in the presence of microbes to ensure the complete eradication of infection from the entire root canal system.

Chlorhexidine (CHX) is widely used in endodontics as an irrigant and intracanal medicaments. CHX has a reasonably wide range of activity against aerobic and anaerobic organisms as well as Candida species. CHX acts by releasing positively charged molecule to allow CHX molecule to penetrate into the microorganism cell, resulting in cell death [10]. Chlorhexidine was demonstrated to be highly efficient against *C. albicans* when compared to calcium hydroxide, especially in gel formulations at 2% concentration [12].

Hoshino *et al.* introduced the use of triple antibiotic paste, which is a mixture of ciprofloxacin, metronidazole and minocycline. Studies have shown that topical application of this paste disinfects the dentinal lesions and recalcifies the softened dentin [13-15]. These antibiotics, in combination, have been reported to be able to penetrate through dentinal tubules and eradicate anaerobic, Gram-positive and Gram-negative microorganisms [16,17].

Propolis, a strongly adhesive resinous compound produced by *Apis mellifera* L. bees, demonstrated antimicrobial activity and used in treatment of bacterial, fungal and inflammatory diseases [18]. The major chemical component present in propolis are flavonoids, phenolics, and various aromatic compounds. Flavonoids are well- known plant compounds that have antioxidant, anti- bacterial, antifungal, antiviral, and anti-inflammatory properties [19,20]. Propolis is well known for its antifungal activities against *C. albicans*[21-26]. However, its effect against *C. albicans* in the dentinal tubules is scarcely studied.

Therefore, the purpose of this ex vivo study was to evaluate and compare the antifungal effectiveness of propolis, triple antibiotic paste, 2% chlorhexidine gel, and calcium hydroxide with propylene glycol against *C. albicans*.

## Methods

The investigation protocol was approved by the Research and Ethics Committee of International Medical University. The method used was a modification of the one previously described [27].

### Microorganism

Freshly sub cultured (24 hours) *C. albicans* were used throughout this study. The *C. albicans* were grown in Sabouraud Dextrose (SD) Agar and SD Broth (BD Difco™, Franklin Lakes, New Jersey, USA).

### Dentine block specimens

A total of 90 intact freshly extracted human teeth, including maxillary central incisors, maxillary lateral incisors, maxillary canines and mandibular canines with complete root formation were selected for this study. The teeth were cleaned with periodontal curettes to remove periodontal tissues and bone and stored in saline. A low-speed diamond edge-coated disc (Bredent®, Wittighausen, Senden, Germany) mounted on a milling machine under water cooling was used to section the teeth below the cementoenamel junction and the apical part of the root to obtain 6 mm of the middle third of the root. Root cementum was removed using long cylindrical diamond burs, in a high-speed handpiece (Kavo®, Charlotte, North Carolina, USA), under water cooling to obtain a dentine block. Pesso Reamer no. 2 (Mani®, Utsunoniya, Tochigi, Japan) in a low-speed handpiece (Kavo, Charlotte, North Carolina, USA) was used to standardize the internal diameter (0.9 mm) of root canals. The dentine blocks were subjected to ultrasonic irrigation (EndoActivator, Dentsply, Weybridge, Surrey, UK) using 5.25% sodium hypochlorite (Clorox®, Oakland, California, USA) and then 17% EDTA (Calasept®, Nordiska Dental, Ängelholm, Skåne Country, Sweden) for one minute to remove smear layer. The dentine blocks were thoroughly rinsed with sterile saline after each irrigation. Next, the dentine blocks were subjected to sterilization by autoclave (LTE®, Oldham, Lancashire, UK) for 20 minutes at 121°C.

The outer surfaces of the specimens were covered with nail varnish to prevent contact of the *C. albicans* medicament with the external surface. Ten petri dishes containing wax with a flat surface were prepared, and surface sterilized using 70% ethanol and air dried in a sterile biosafety cabinet before use. The dentine blocks were up righted, and the apical ends were fixed to the petri dishes with wax, with a thin small square of sterilized plastic strip obliterating the apical orifice to prevent any softened wax from entering the canals.

### Inoculation of dentinal blocks with *C. albicans*

*C. albicans* were suspended in 20.0 ml of SD broth. The cell suspension was adjusted to match the turbidity of 1.5 × 10 [8] CFU mL^-1^ (equivalent to 0.5 McFarland standards). The fungal inoculum was transferred into the dentine blocks with the use of sterile 5.0 mL syringes (Terumo®, Somerset, New Jersey, USA) with 30-gauge needles (Terumo, Somerset, New Jersey, USA) in a sterile laminar flow hood. The coronal part of the dentine blocks were then sealed immediately using Parafilm (Parafilm M®, Brand, Wertheim, Baden-Württemberg, Germany). The dentine blocks were incubated at 37°C for 21 days, with renewal of *C. albicans* every 3 days.

### Placement of intracanal medicaments

Following the inoculation period, the canal of dentine blocks were irrigated with sterile saline and dried with sterile paper points. The 90 dentine blocks were divided into five groups, according to the intracanal medicament used, as follows:

• Group I (20 specimens): 95% propolis (Stakich, Royal Oak, Michigan, USA) was mixed with saline in a ratio of 1.5:1 (wt/vol) to obtain a paste like consistency.

• Group II (20 specimens): Mix of equal weight (1:1:1) of ground metronidazole (UPL, Ankleshwar, Gujarat, India), ciprofloxacin (Apex Pharmacy, Petaling Jaya, Selangor, Malaysia) and minocycline (YSP, Kuala Lumpur, Selangor, Malaysia) was mixed with sterile saline in a ratio of 1.5:1 (wt/vol) to obtain a paste like consistency.

• Group III (20 specimens): 2% chlorhexidine gel (Consepsis V®, Ultradent, South Jordan, Utah, USA).

• Group IV (20 specimens): non-setting Ca(OH)_2_ (Produits Dentaires SA, Vevey, Vaud, Switzerland) was mixed with propylene glycol in a ratio of 1.5:1(wt/vol) to obtain a paste like consistency.

• Group V (10 specimens) sterile saline as matched untreated control group to provide base line data on fungal growth over time.

Each group was further divided into two subgroups: subgroups I (A1 and A2), subgroups II (B1 and B2), subgroups III (C1 and C2), subgroups IV (D1 and D2), and subgroups V (E1 and E2) according to the experimental periods.

The intracanal medicaments were placed in the canal with the help of sterile 5.0 mL syringes (Terumo®, Somerset, New Jersey, USA) and gel etchant needle tip (Kerr®, Orange, California, USA) until the canals were completely filled. Following the placement of all intracanal medicaments inside the dentinal blocks, the coronal orifices were all sealed with Parafilm (Parafilm M®, Brand, Wertheim, Baden-Württemberg, Germany). The blocks were kept at 37°C according to the experimental periods (1 and 7 days).

### Collection of dentinal shavings

At the end of the experimental periods, the dentine blocks were removed from the petri dishes and the canals were irrigated using sterile saline and the canal walls were cleaned using ultrasonic tips to ensure complete removal of the medicament. The canals were dried with sterile paper points. Samples of dentinal shavings were collected after day 1 medicament for A1, B1, C1, D1 and E1, and after day 7 for A2, B2, C2, D2 and E2.

**Figure 1:**
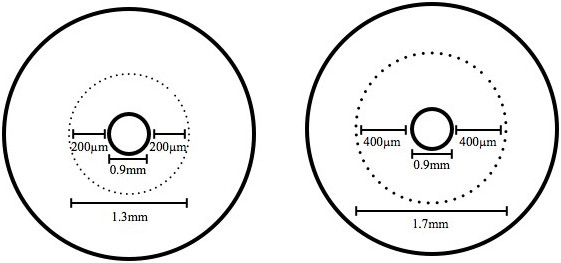
**Schematic presentation of the cross section of root canal dentine and drill size selection.** Pesso Reamer (Mani®, UT, Japan) size no. 4 (1.3 mm diameter) and no. 6 (1.7 mm) allowed a depth of 0.2 mm (200 μm) and 0.4 mm (400 μm) of dentinal shavings to be collected a 0.9 mm diameter canal respectively.

Dentinal shavings were collected using Pesso Reamer (Mani®, Utsunoniya, Tochigi, Japan) size no. 4 (1.3 mm diameter) followed by size no. 6 (1.7 mm diameter), in a low speed handpiece (Kavo®, Charlotte, North Carolina, USA). These sizes of the pesso reamers allow a depth of 200 μm and 400 μm of dentinal shavings to be collected (Figure 1). Dentinal shavings were transferred immediately into a micro-centrifuge tube (Axygen®, Corning, Tweksburry, Massachusets, USA) containing 1.0 ml sterile SD broth.

### Antimicrobial assessment

A sterile micro tip was used to take 0.1 ml of broth containing dentinal shavings, transferred to another tube containing 0.9 ml sterile SD broth. The content of each tube was serially diluted from 10^-1^ until 10^-7^. 300 μl of the diluted shavings was spread evenly using a L-shaped glass rod and triplicated on three occasions. These plates were incubated for 24 hours at 37°C and the colonies were counted and readings were tabulated.

### Statistical analysis

The SPSS computer software version 18.0 (SPSS Inc., Chicago, Illinois, USA) was used to perform statistical analysis. The values were analysed using non-paramatric Kruskal-Wallis and Mann–Whitney-U tests to compare the median reduction of *Candida albicans* between all intracanal medicaments. Probability values of P < 0.05 were set as the reference for statistically significant results.

## Results

The control group showed viable *C. albicans* at all experimental times, confirming the efficiency of the methodology.

Non-parametric Kruskal Wallis test showed significance differences between the intracanal medicaments and saline on both days and at different depths (Table 1).

**Table 1:** Comparison of CFUs between the tested medicaments and saline

	**N**	**Day 1**				**Day 7**			
		**200 μm**		**400 μm**		**200 μm**		**400 μm**	
		**Median**	**Q**_**3**_**-Q**_**1**_	**Median**	**Q**_**3**_**-Q**_**1**_	**Median**	**Q**_**3**_**-Q**_**1**_	**Median**	**Q**_**3**_**-Q**_**1**_
Saline	5	3.49×10^7^	1.90×10^7^	3.40×10^5^	2.46×10^5^	2.08×10^7^	8.95×10^6^	3.06×10^5^	96500.00
Propolis	10	4695.00	4.95×10^5^	1.15×10^5^	5.33×10^5^	896.00	53835.21	8426.67	15389.54
TAP	10	1502.25	3520.61	1052.63	13013.58	28.75	24.13	59.25	153.21
CHX	10	4.25	172.83	3.25	76.67	21.75	101.13	1.00	25.00
Ca(OH)_2_-PG	10	865.00	2107.88	207.83	461.91	29.25	423.42	6.25	107.75

Median and Interquartile range of all the groups are also shown.

Table 2 shows the median and percentage difference difference in CFU between various intracanal medicaments. When the results were compared to the untreated control group (sterile saline), the p-value shown could be attributed directly to the effect of the medicaments tested.

**Table 2:** Mann–Whitney U-test to study the difference between various intracanal medicaments and CFU

	**Day 1**				**Day 7**			
	**200 μm**		**400 μm**		**200 μm**		**400 μm**	
	**Median reduction in CFU(%)**	**p value**	**Median reduction in CFU(%)**	**p value**	**Median reduction in CFU(%)**	**p value**	**Median reduction in CFU(%)**	**p value**
Saline vs Propolis	3.49x10^7^ (99.98%)	0.003	2.25×10^5^ (66.18%)	0.096	2.08×10^7^ (99.99%)	0.003	2.98×10^5^ (97.25%)	0.003
Saline vs TAP	3.49x10^7^ (99.99%)	0.003	3.39×10^5^ (99.69%)	0.013	2.08×10^7^ (99.99%)	0.003	3.06×10^5^ (99.98%)	0.003
Saline vs CHX	3.49x10^7^ (99.99%)	0.003	3.40×10^7^ (99.99%)	0.003	2.08×10^7^ (99.99%)	0.003	3.06×10^5^ (99.99%)	0.003
Saline vs Ca(OH)_2_-PG	3.49x10^7^ (99.99%)	0.003	3.40×10^5^ (99.99%)	0.003	2.08×10^7^ (99.99%)	0.003	3.06×10^5^ (99.99%)	0.003
Propolis vs TAP	3192.75 (68%)	0.102	1.14×10^5^ (99.08%)	0.021	867.25 (96.79%)	0.001	8367.42 (99.30%)	0.001
Propolis vs CHX	4690.75 (99.91%)	0.001	1.15×10^5^ (99.99%)	0.000	874.25 (97.57%)	0.003	8425.67 (99.99%)	0.000
Propolis vs Ca(OH)_2_-PG	3830.00 (81.58%)	0.047	1.15×10^5^ (99.82%)	0.001	866.75 (96.74%)	0.012	8420.42 (99.93%)	0.001
TAP vs CHX	1498.00 (99.71%)	0.004	3347.78 (95.09%)	0.001	7.00 (24.34%)	0.965	58.25 (98.31%)	0.004
TAP vs Ca(OH)_2_-PG	637.25 (42.42%)	0.847	844.80 (80.24%)	0.027	0.50 (1.71%)	0.627	50.00 (84.39%)	0.102
CHX vs Ca(OH)_2_-PG	860.75 (99.51%)	0.002	204.58 (98.44%)	0.004	7.50 (25.64%)	0.354	5.25 (84.00%)	0.157

*p-value < 0.05 was considered as statistically significant.

***p-value < 0.005 was considered as highly significant.

### Fungal reduction at 200μm dentinal tubule depth

**Figure 2:**
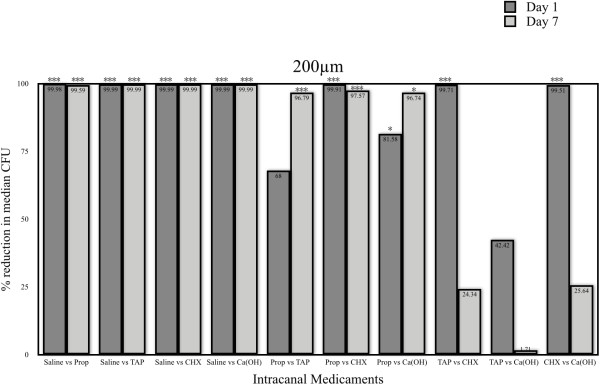
**Comparison of percentage reduction in median CFU intracanal medicaments at 200 μm dentinal tubule depths on day 1 and day 7.** (p value < 0.05 = *, p < 0.01 = **, p < 0.001 = ***).

On the day 1, all medicaments showed highly significant fungal reduction (p < 0.005) when compared to the control (sterile saline). When the four medicaments were compared to each other, CHX showed the best antifungal activity (p < 0.005), followed by Ca(OH_)2_ with propylene glycol which was better than propolis (p < 0.005) but no difference to TAP (p > 0.05). TAP showed no significant difference compared to propolis (p > 0.05) (Figure 2).

At day 7, all medicaments showed highly significant fungal reduction (p < 0.005) when compared to the control (sterile saline). When the four medicaments were compared to each other, CHX showed highly significant difference only with propolis (p < 0.005) with no significant difference to Ca(OH)_2_ with propylene glycol and TAP. Ca(OH)_2_ with propylene glycol showed significant difference with propolis (p < 0.05) with no significant difference with TAP (p > 0.05). TAP also showed highly significant difference to propolis (p < 0.005) (Figure 2).

### Fungal reduction at 400μm dentinal tubule depth

**Figure 3:**
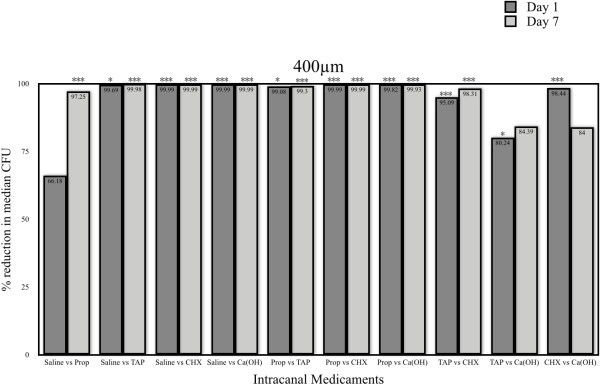
**Comparison of percentage reduction in median CFU intracanal medicaments at 400 μm dentinal tubule depths on day 1 and day 7.** (p value < 0.05 = *, p < 0.01 = **, p < 0.001 = ***).

At day 1, when compared to the control (sterile saline), all medicaments showed highly significant fungal reduction (p < 0.005) except propolis (p > 0.05). When the four medicaments were compared to each other, CHX was the best with a highly significant difference compared to other three medicaments (p < 0.005). Ca(OH)_2_ with propylene glycol showed significantly better results than to TAP (p < 0.05) and highly significant to propolis (p < 0.005). TAP was significantly better than propolis (p < 0.05) (Figure 3).

At day 7, when compared to the control (sterile saline), all medicaments showed highly significant fungal reduction (p < 0.005). When the four medicaments were compared to each other, CHX showed highly significant difference (p < 0.005) to TAP and propolis but no significant difference to Ca(OH)_2_ with propylene glycol (p > 0.05). Ca(OH)_2_ with propylene glycol and TAP showed highly significant difference to propolis (p < 0.005) but no significant difference among each other (p > 0.05) (Figure 3).

## Discussion

The use of a biocompatible intracanal medicament with antimicrobial properties between appointments may completely eradicate the micro-organisms from the root canal system and could significantly increase the success of root canal treatment [28]. The in vitro model developed by Haapasalo & Orstavik has been used to assess the efficacy of endodontic medicaments in the disinfection of dentinal tubules [27]. In the present study, this model was modified to include natural human teeth as specimens, thereby provided a better simulation to the clinical settings. Sectioning and shaping of the canal were maintained to a standard of 6.0 mm in height and 0.9 mm in diameter to ensure placing an uniform quantity of fungi during inoculation, and intracanal medicaments. Also, quantitative analysis of fungi in the dentine tubules was done to define a percentage of reduction in CFU in infected dentine before and after the application of intracanal medicaments. *C. albicans* was chosen due to the presence of the organism in 7-18% cases of persistent apical periodontitis [4].

In the present study, 2% chlorhexidine gel was effective for *C. albicans* at dentinal tubule depths of 200 μm and 400 μm on day 1 and day 7 the fungicidal inhibition were found to be statistically significant. The possible reason could be that, the bactericidal dosage of 2% and gel formulation helps to retain the CHX in close proximity to the root canal walls and dentinal tubules [29,30]. The CHX innate substantive properties inhibits re-infection for a duration of at least 12 weeks [31,32]. The result of the present study was similar to that of Vaghela *et al.*, who showed that CHX had the highest fungicidal activity at 200 μm and 400 μm [12]. However, CHX should be used cautiously when sodium hypochlorite (NaOCl) was used as a root canal irrigant previously. This is because the reaction between NaOCl and CHX leads to the formation of an orange-brown precipitate, resulting in a chemical smear layer that covers the dentinal tubules and may interfere with the seal of the root canal filling. In addition, this precipitate may be cytotoxic to periapical tissues. The other limitation includes the cytotoxic effect with live tissues for extended periods, as demonstrated in the literature [33]. Therefore, thorough flushing of root canal is mandatory before using CHX as an intracanal medicament.

The fungicidal effect of propolis was statistically significant in eliminating *C. albicans* at dentinal tubule depths of 200 μm on day 1 and at both depths on day 7. However, the reduction was not statistically significant at the depth of 400 μm on day 1. The possible reason for lower antifungal activity of propolis on day 1, at the depth of 400 μm could be due to the slower rate of penetration in the dentinal tubules. The diffusing property of the medicaments could be improved by adding vehicles such as propylene glycol. The antifungal action of propolis is due to the presences of flavonoids and phenolic acids which interact with the cellular sulphydryl compounds on the cell wall. This damages the integrity of the yeast cell wall and results in detachment of fungal cell wall and reduction of germ tube formation and hyphal length. As a result, it inhibits yeast-mycelial conversion which ultimately prevents cell division [22,23]. In addition, the anti-inflammatory and anti-oxidant properties of propolis could further enhance the healing of the periapical tissue. However, the application of propolis should be avoided in patients who are known to be allergic to pollen [34].

Calcium hydroxide with propylene glycol resulted in statistically significant inhibition of *C. albicans* at dentinal tubule depths of 200 μm and 400 μm at day 1 and day 7. Calcium hydroxide reacts by releasing hydroxyl ions leading to a highly alkaline environment which microorganisms cannot survive. Chemically it damages the microbial cytoplasmic membrane, suppresses enzyme activity and disrupts the cellular metabolism or microorganisms [8]. Calcium hydroxide is the most widely used intracanal medicaments, mainly in teeth with apical periodontitis. It has several beneficial properties, such as antimicrobial effect, tissue compatibility, ability to induce the formation of mineralized tissue, inactivation of bacterial endotoxin and ability to promote repair. However, *C. albicans* have been shown to be resistant to it [7]. In the present study, calcium hydroxide was effective which probably is due to the addition of a vehicle, namely propylene glycol, which allows the release of hydroxyl ions for a longer period, and enhances the diffusibility of calcium hydroxide into the dentinal tubules [35]. The result of the present study was similar to the study of Vaghela *et al*., demonstrated that calcium hydroxide with propylene glycol had high antifungal activity at 200 μm and 400 μm [10]. However, calcium hydroxide in its high pH may cause necrosis of the surrounding tissue, and long term use may increase brittleness of root dentin which increases the risk of future cervical root fractures [36].

Triple antibiotic paste delivered statistically significant inhibition of *C. albicans* at dentinal tubule depths of 200 μm and 400 μm at day 1 and day 7. Triple antibiotic paste contains minocycline which inhibits protein synthesis on the surfaces of ribosome which might be the reason for the antifungal property, while metronidazole and ciprofloxacin can help fibroblasts to generate the synthesis of extracellular matrix and collagen and contribute to the development of structural framework [16,37]. Triple antibiotic paste (TAP) was able to penetrate into dentinal tubules and proved effective against all microorganisms, anaerobic, gram-positive and gram negative microorganisms [15]. TAP promotes healing, repairs periapical tissue besides creating an aseptic environment and accelerates functional development of the pulp-dentin complex [38]. However, care should be taken in patients who are known to be allergic to tetracycline, and also in anterior teeth where discolouration could be caused by minocycline [36].

## Conclusions

Propolis was less effective than triple antibiotic paste, 2% chlorhexidine gel and calcium hydroxide with propylene glycol against *C. albicans* on day 1 at 400 μm deep inside the dentinal tubules, but equally effective after 7 days at both depths.

## Competing interests

The authors declare that they have no competing interests.

## Authors’ contributions

EGC carried out all the methods, data analysis and drafted the manuscript. AP designed the study, acted as a demonstrator to carry out the ‘dental part’ of methods, involved in revising the manuscript critically, PA supervised the whole progress of study, AP contributed in data analysis and manuscript editing. FDA has been involved in designing the study, in the microbiology part of the study and manuscript editing. All authors read and approved the final manuscript.

## Authors’ information

Dr Eu Gene Chua, BDS (IMU). Dr Abhishek Parolia, BDS (Hons) (Manipal AHE), MDS (Manipal AHE). Dr Priya Ahlawat, BDS (Manipal AHE), MMedSc (Restorative) (Sheff). Prof Dr Allan Pau, BDS (Lond), DDPH RCSEng, MSc (Lond), PhD (Lond), FDS RCSEd. Dr Fabian Davamani Amalraj, MSc (Madr), PhD (Madr).

## Pre-publication history

The pre-publication history for this paper can be accessed here:

http://www.biomedcentral.com/1472-6831/14/53/prepub
